# Positive selection in glycolysis among Australasian stick insects

**DOI:** 10.1186/1471-2148-13-215

**Published:** 2013-09-30

**Authors:** Luke T Dunning, Alice B Dennis, Geoffrey Thomson, Brent J Sinclair, Richard D Newcomb, Thomas R Buckley

**Affiliations:** 1 Landcare Research, Private Bag 92170, Auckland, New Zealand; 2 School of Biological Sciences, University of Auckland, Auckland, New Zealand; 3 Allan Wilson Centre for Molecular Ecology and Evolution, Auckland, New Zealand; 4 Department of Biology, The University of Western Ontario, London, ON, Canada N6G 1L3; 5 The New Zealand Institute for Plant & Food Research Limited, Auckland, New Zealand; 6 Imperial College London, Silwood Park Campus, Buckhurst Road, SL5 7PY, Ascot, Berks, UK

**Keywords:** Lanceocercata, Molecular evolution, Lipid biosynthesis, Oxidative stress, Phosphoglucose isomerase

## Abstract

**Background:**

The glycolytic pathway is central to cellular energy production. Selection on individual enzymes within glycolysis, particularly phosphoglucose isomerase (*Pgi*), has been associated with metabolic performance in numerous organisms. Nonetheless, how whole energy-producing pathways evolve to allow organisms to thrive in different environments and adopt new lifestyles remains little explored. The Lanceocercata radiation of Australasian stick insects includes transitions from tropical to temperate climates, lowland to alpine habitats, and winged to wingless forms. This permits a broad investigation to determine which steps within glycolysis and what sites within enzymes are the targets of positive selection. To address these questions we obtained transcript sequences from seven core glycolysis enzymes, including two *Pgi* paralogues, from 29 Lanceocercata species.

**Results:**

Using maximum likelihood methods a signature of positive selection was inferred in two core glycolysis enzymes. *Pgi* and Glyceraldehyde 3-phosphate dehydrogenase (*Gaphd*) genes both encode enzymes linking glycolysis to the pentose phosphate pathway. Positive selection among *Pgi* paralogues and orthologues predominately targets amino acids with residues exposed to the protein’s surface, where changes in physical properties may alter enzyme performance.

**Conclusion:**

Our results suggest that, for Lancerocercata stick insects, adaptation to new stressful lifestyles requires a balance between maintaining cellular energy production, efficiently exploiting different energy storage pools and compensating for stress-induced oxidative damage.

## Background

Respiration generates the cellular energy required for the fundamental biological processes of growth, development and reproduction. The pathways involved in respiration are well characterised, flowing from glycolysis to the Krebs cycle, along with well-understood branch points, including the pentose phosphate pathway activated during periods of oxidative stress [1] and increased lipid biosynthesis [2]. Nonetheless, how these pathways as a whole evolve to allow organisms to live in different environments and adopt new life history traits that might require altered energy levels requires further attention [3].

Enzymes in metabolic pathways are kinetically entwined in a complex conveyer belt of substrate to end-product [4]. As a result of this interdependence, mutations that alter *in vitro* catalytic activity may not alter the observed *in vivo* phenotype [4]. Enzymes with the greatest flux control through pathways are likely targets of natural selection [5-7], due to the probability of fixation of any adaptive change associated to its conferred advantage. Flux control may be proportionally distributed across the pathway [4] or asymmetrically localised in enzymes at the top of the pathway [8,9]. The evolution and incidence of selection is dependent on the properties and complexities of the pathway, with the disparity between current and optimal flux being a key factor [9]. The evolution of flux control and selection on enzyme polymorphisms has been intensively studied in the glycolytic pathway of *Drosophila*[3,6,10,11].

Glycolysis comprises a series of ten enzyme-catalysed reactions that convert glucose to pyruvate, the substrate for the Krebs cycle. Concurrently, glycolysis also generates small quantities of ATP and NADH that are utilised by downstream high-energy yielding processes. Although glycolysis is an essential biological pathway conserved across all eukaryotes, the component enzymes exhibit extensive variation at both the nucleotide and protein levels, which may be the target of selection [3]. In *Drosophila*, positive selection is centred on the glucose-6-phosphate intersection for glycolysis and the pentose phosphate pathway [11]. The branch point enzymes are hypothesised to possess novel flux control resulting in higher selection coefficients for adaptive mutations [3]. In the *Drosophila* species studied, branch point enzymes accumulate more adaptive mutations than enzymes at the top of the pathway, which are conserved by strong purifying selection [10]. Simulations provide theoretical support for this empirical evidence, and make the prediction that the further flux is from optimum, the more likely adaptive substitutions are to occur disproportionately in branching enzymes [9]. Further work is required in species with diverse metabolic demands to test the generality of the *Drosophila* findings [3]. Positive selection and adaptation within glycolysis and its connecting pathways are likely to be a balance between maintaining cellular energy production, reducing the associated damage caused by oxidative stress [1], and efficiently exploiting the available substrate storage pools (e.g. glycogen or triglycerides) [6].

A majority of evidence for selection within the glycolytic enzymes, and its correlation with fitness, has been accumulated for the dimeric enzyme phosphoglucose isomerase (PGI; EC 5.3.1.9), which is not a rate-limiting step in glycolysis [10]. Variation in *Pgi* has been associated with flight performance, thermal adaptation and other fitness-related traits in a diverse array of organisms [12,13], including arthropods [14-18], bacteria [19], molluscs [20], and plants [21]. PGI is thought to be under climate-related directional selection in the beetle *Chrysomela aeneicollis* (Coleoptera), where distinct alleles with functional and physiological differences are restricted to cold or warm habitats [15]. Comparisons between *Colias* and *Melitaea cinixia* butterfly *Pgi* nuclear polymorphisms indicate convergent evolution [18]. Increased *Colias* survival [14] and *M. cinixia* population growth rates [16] have both been correlated with *Pgi* allelic variants. This variation is likely a product of long-term balancing selection on charge-changing amino acid substitutions on external loops of the protein’s surface [17,18]. The consistent association of *Pgi* polymorphisms with variation in individual performance, coupled with no known duplication events in arthropods, has led to this locus being proposed as a general adaptive marker for conservation genetics in this phylum, akin to MHC in vertebrates [13].

Flight [22] and adaptation to low temperature [23] are both linked with varying energy demands and increased oxidative stress. The metabolic rate of insects during flight may increase by over a 100-fold compared with when they are at rest [22]. This metabolically-demanding activity results in increased oxidative stress, which causes cellular damage and even increased mortality in houseflies (*Musca domestica*) [24]. Furthermore, the increased energy demand of flight may require the insect to exploit different fuels, as seen in the flight-capable morphs of the wing-polymorphic cricket (*Gryllus firmus*), where winged individuals increase lipid biosynthesis in preparation for flight [25]. Similar patterns are observed in insects exposed to low temperatures, where depressed metabolic rates result in the accumulation of reactive oxygen species [23] and there is increased lipid biosynthesis in preparation for overwintering in several insect species [26]. One way to test the impact of varying metabolic demands on the evolution of glycolysis is to study variation in these component genes across related taxa that have evolved to new, potentially stressful lifestyles, and that vary in their ability to fly.

The Australasian Lanceocercata stick insects (Order: Phasmatodea) form a monophyletic clade [27-30] comprising species from five subfamilies, Pachymorphinae, Tropidoderinae, Xeroderinae, Phasmatinae, Eurycanthinae, and Platycraninae, with distinct morphological characters [28]. The Lancerocercata have undergone an impressive evolutionary radiation over the last 50 Mya [29]. This includes the convergent evolution of different body forms [30], loss of flight in one or both sexes, shifts in diet, and adaptation to low-temperature environments in New Zealand, including four wingless species found in high alpine areas where they regularly experience sub-zero temperatures [31,32]. These contrasting lifestyles within Lanceocercata are expected to afford a diverse range of metabolic demands. This diversity provides a comprehensive model system in which to determine whether glycolysis has been the target of positive selection, and if so, at which steps and even at which sites within these enzymes selection has occurred. To pinpoint positive selection within Lanceocercata we obtained partial cDNA sequences of seven core glycolytic enzymes, together with the enzymes immediately before and after the pathway, across a phylogeny of 29 Lanceocercata species. We found that the *Pgi* gene has been duplicated within Lanceocercata, with at least two of the three paralogous copies and *Gapdh* evolving under positive selection.

## Results

### DNA sequencing of genes from glycolysis

**Figure 1:**
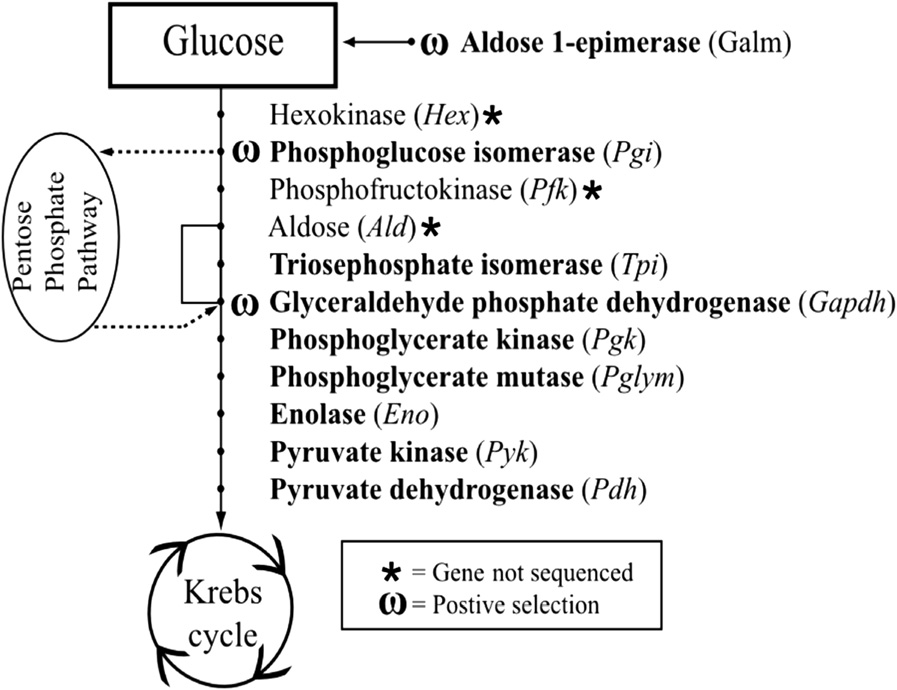
**Positive selection in the glycolytic pathway.***Galm* and *Pdh* are not part of the core glycolysis genes but operate in the steps immediately before or after the pathway. Genes in Bold were sequenced in this study, with ω denoting positive selection having been inferred at individual codons. Branch points to and from the pentose phosphate pathway are included.

We used *de novo* transcriptomes derived from 454 sequencing of three New Zealand species (*Micrarchus* nov. sp. 2 [31], *Niveaphamsa annulata* and *Clitarchus hookeri*) to design PCR primers and amplify 19 genes to reconstruct the evolutionary history of 29 Lanceocercata stick insects. Of these partial gene transcripts, ten were distributed across the glycolytic pathway, including seven of the ten core enzymes (Figure 1). In addition, six nuclear protein coding genes with a variety of cellular functions (*Calr*, *Ef1a*, *Flnc*, *Gsk3b*, *Mer* and *Sord*), two mitochondrial genes (*COI*, *COII*), and 28S rDNA were sequenced from cDNA for phylogenetic reconstruction.

### Pgi duplications

Although only a single copy of *Pgi* has been previously characterised in arthropods, the transcriptome assemblies revealed three paralogous copies of *Pgi* within Lanceocercata, with the duplication events presumably occurring after the split between *Timema* and all other Phasmatodea (Additional file 1: Figure S1), which occurred more than 95 Myr ago [29]. Sequences of the full length open reading frame from the transcriptomes show extensive predicted amino acid variation within and among *Pgi* paralogues (Additional file 1: Figure S2), with the percentage of identical amino acid sites within the three species being higher for pairwise comparisons among orthologues (mean = 94.2%) than paralogues (mean = 70.2%). Subsequent PCR amplification of *Pgi* paralogues across a wider range of species was possible from a region within *Pgi1* (675 bp) and *Pgi2* (1452 bp), but not from *Pgi3* due to the lack of unique primer binding sites.

### Lanceocercata phylogeny reconstruction

**Figure 2:**
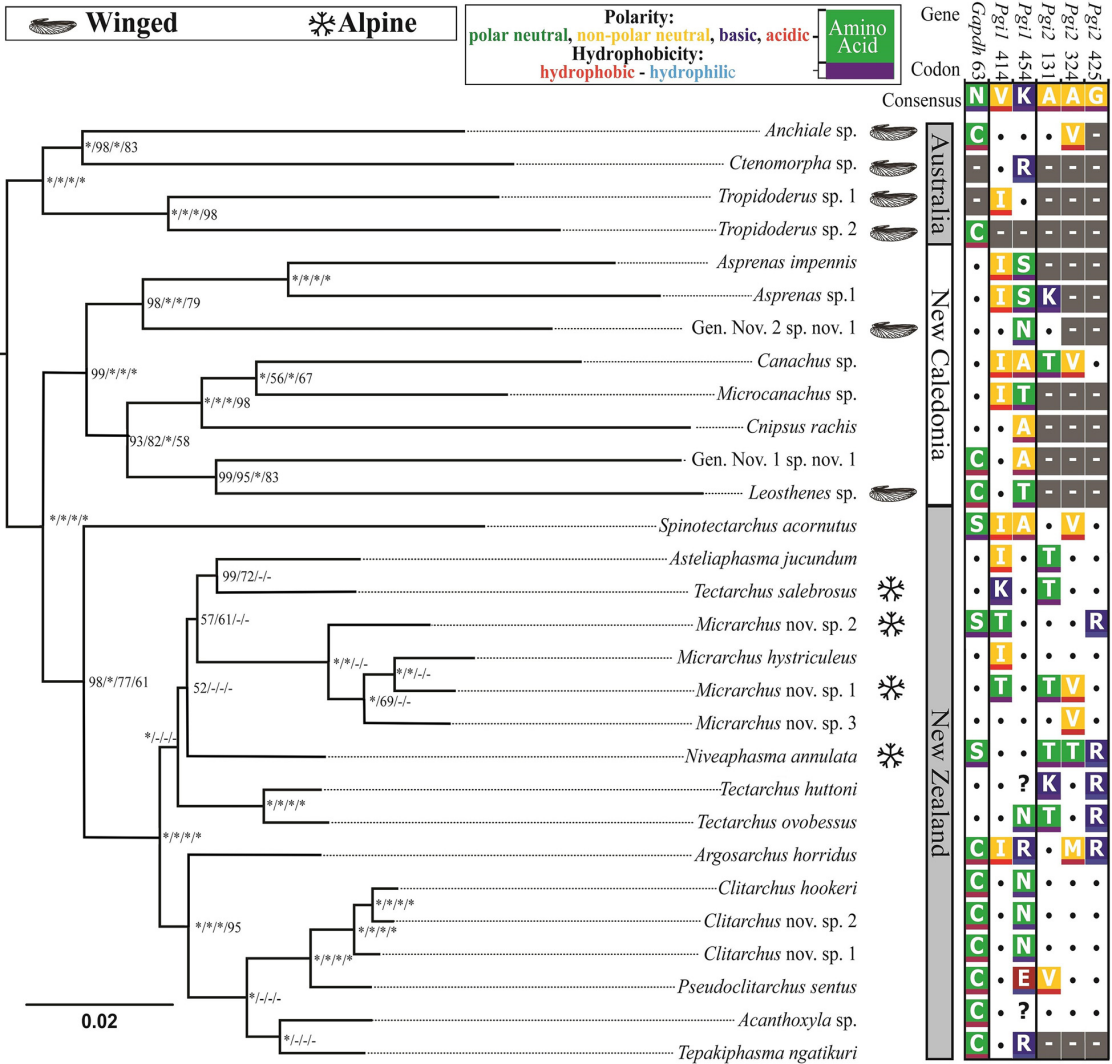
**Lanceocercata phylogeny with codons under selection.** Combined Bayesian Lanceocercata phylogenetic tree constructed using 15,077 bp from 19 genes with branch lengths proportional to the estimated number of substitutions per site. Nodal support values are nucleotide posterior probabilities/ nucleotide maximum likelihood bootstraps/ amino acid posterior probabilities and /amino acid maximum likelihood bootstraps. Asterisks denote nodes with 100% support; Dashes indicate nodes with less than 50% support. Amino acids sites identified by the M8 Bayes Empirical Bayes (BEB) analysis are depicted on the right with polarity and hydrophobicity highlighted.

To infer evolutionary relationships among the sampled Lanceocercata species, a combined Bayesian phylogeny (Figure 2) was constructed from a 15,034 bp partitioned alignment of the 18 protein coding genes and 28S. An average of 13,990 bp of sequence data was used per species, with the minimum of 9,692 bp (Gen. Nov. 1, sp. nov. 1) and a maximum of 15,034 bp (*Acanthoxyla* sp. *Argosarchus horridus*, *Asteliaphasma jucundum*, all *Clitarchus* sp., *Pseudoclitarchus sentus*, *Tectarchus ovobessus* and *T. salebrosus*). This combined phylogeny has variable support for a monophyletic clade containing all New Zealand species. The different lifestyles of flight and alpine habitation are scattered across the phylogeny.

### Codons under positive selection in the glycolytic pathway

The selective pressures acting on a protein-coding DNA sequence can be inferred from the ratio of nonsynonymous to synonymous nucleotide substitutions (ω). Purifying selection is indicated by ω <1, whereas ω =1 implies neutral evolution and ω >1 indicates positive selection [33]. Typically, selection acts on relatively few amino acid sites within a protein-coding gene. As a result, standard neutrality tests that average ω across the entire reading frame often fail to infer incidences of positive selection [34]. Amino acid residues likely to have been influenced by positive selection within a protein can be identified by estimating ω with site-based maximum likelihood methods [35], even though this approach may be overly conservative [36]. Positive selection can be inferred by comparing the goodness of fit for a set of nested models. Models that allow ω to vary above one to include the effect of positive selection are compared with null models where ω is not allowed to fluctuate above 1, meaning the observed nucleotide variation is solely a product of purifying selection and neutrality. Likelihood ratio tests and chi-square distributions are used to assess the statistical significance between the models.

Site-based likelihood models were used to infer positive selection acting on ten Lanceocercata genes associated with the glycolytic pathway (Figure 1). We found that all glycolysis genes are predominantly evolving under the influence of strong purifying selection when a single value of ω is calculated across the protein coding sequences using the one-ratio (M0) model (mean ω = 0.10; range 0.04-0.19; Table 1). However, there is significant among-site variation in ω within all genes, with the M3 model permitting three ω values, providing a significantly better fit to the data than the M0 model (*p* value <0.001*;* d.f. = 4; M0:M3; Table 1; Additional file 1: Table S1). To test for positive selection the M7 beta neutral model with eight categories of ω, all less than one and together comprising a beta distribution, is compared with the M8 model that has one additional ω value that is free to vary above one and therefore account for positive selection. The M8 model is a significantly better fit for three core glycolysis genes (*Pgi1* (*p* < 0.001), *Pgi2* (*p* < 0.001), *Gapdh* (*p* < 0.05) and aldose 1-epimerase (*Galm* (*p* < 0.05); d.f. = 2)*,* which links glycolysis to the Leloir pathway, indicating positive selection acts on these genes (Table 1; Additional file 1: Table S1). A more conservative likelihood ratio test for positive selection replaces the M7 null model with M8a, which has an extra category of ω fixed at one. The M8:M8a nested model comparison identifies positive selection in *Pgi1* (*p* < 0.001) and *Pgi2* (*p* < 0.05) (d.f. = 50:50 mixture of point mass 0 and 1; Additional file 1: Table S1). The most stringent approach to identify positive selection compares the M1a null model, which has two values of ω (ω <1, ω =1), and M2a, which has an additional third category of ω that is greater than one. This approach still identifies positive selection in *Pgi1* (*p* < 0.001; d.f. = 2; Additional file 1: Table S1), suggesting that this inference is especially robust.

**Table 1:** Positive selection in the glycolysis enzymes from Lanceocercata stick insects

**Gene**	**pp**	**n (m)**	**bp**	**ө**	**k**	**ω (M0)**	**M0:M3**	**M7:M8**	**M8:M8a**
*Galm*	−1	28 (2)	681	0.11	1.86	0.16	210.93**	6.08*	0.68
*Pgi1*	2	20 (3)	1452	0.06	2.80	0.10	292.65**	21.92**	15.33**
*Pgi2*	2	28 (4)	675	0.08	4.69	0.10	216.97**	16.55**	4.59*
*Tpi*	5	28 (1)	651	0.09	2.37	0.14	53.06**	1.27	0.95
*Gapdh*	6	29 (2)	837	0.09	2.60	0.04	126.85**	10.78*	0.24
*Pgk*	7	29 (1)	915	0.13	2.70	0.19	283.07**	3.34	0.57
*Pglym*	8	27 (0)	486	0.10	3.67	0.08	153.05**	2.10	0.68
*Eno*	9	25 (1)	903	0.09	1.99	0.11	131.51**	1.89	0.01
*Pyk*	10	29 (2)	777	0.10	3.07	0.04	146.00**	0.00	0.00
*Pdh*	+1	27 (2)	729	0.09	3.95	0.05	103.29**	0.46	0.00

Selection inferred using likelihood ratio tests between nested site-specific models.

*Eno =* Enolase; *Galm* = Aldose 1-epimerase; *Gapdh* = Glyceraldehyde 3-phosphate dehydrogenase; *Pdh* = Pyruvate dehydrogenase; *Pgi1* = Phosphoglucose isomerase 1; *Pgi2* = Phosphoglucose isomerase 2; *Pgk* = Phosphoglycerate kinase; *Pglym* = Phosphoglycerate mutase; *Pyk* = Pyruvate kinase; *Tpi* = Triosephosphate isomerase; PP = pathway position of the enzyme out of the ten key glycolysis reactions, N.B. *Galm* and *Pdh* are the steps immediately before and after the pathway. n = number of species with complete and partial (m) sequences; bp = sequence length; ө = nucleotide diversity; k = ratio of transitions to transversions; ω(M0) = ratio of synonymous to nonsynonymous substitutions under the M0 model; 2∆l is given for each model comparison (MO:M3; M7:M8 and M8:M8a), which is twice the difference between the log likelihood of the two nested site-specific models implemented in PAML. Models are judged to have a significantly better fit (* = *P-value* < 0.05; ** = *P-value* < 0.001) based on the χ^2^ distribution with degrees of freedom proportional to the difference in the number of parameters between models; M7/M8 = 2, M8/M8a = 50:50 mixture of point mass 0 and 1.

### Amino acid sites under positive selection in the glycolytic pathway

**Figure 3:**
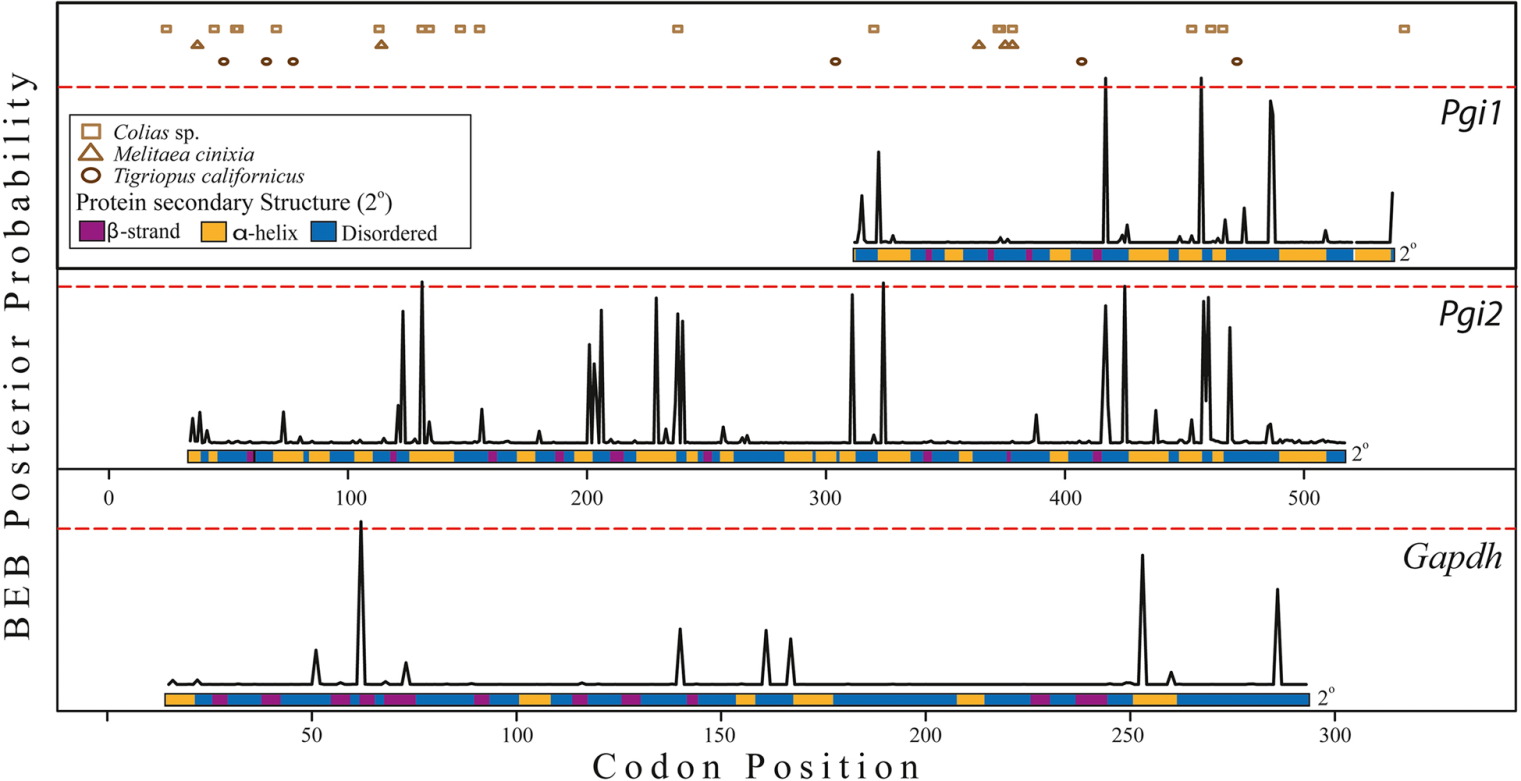
**Positive selection within *Pgi *and *Gapdh*.** Distribution of positively selected sites across three Lanceocercata stick insect genes: phosphoglucose isomerase 1 (*Pgi1*), phosphoglucose isomerase 2 (*Pgi2*) and glyceraldehyde 3-phosphate dehydrogenase (*Gapdh*); Probability of each codon position being under positive selection (ω > 1) using Bayes Empirical Bayes analysis under the M8 site-specific model in PAML. The *Pgi1* analysis consists of 225 codons from 28 species, *Pgi2* 484 codons from 20 species and *Gapdh* 279 codons from 29 species. Codon position based on full-length alignment of both *Pgi* genes from *Micrarchus* nov. sp. 2. Amino acid sites identified in previous studies as either under positive selection, associated with fitness differences or segregating between species for PGI are shown. The secondary structure of each protein in relation to codon position is shown.

Four genes had sites identified under positive selection in the nested M7:M8 site models. A subsequent Bayes Empirical Bayes (BEB) method implemented in PAML was used to assess the significance of the ω ratio at each of these positively selected codon positions. In total six codons were identified as under significant positive selection (Posterior probability (P) > 95%; Table 2). These include one site in *Gapdh* (Asn63), two in *Pgi1* (Val414 and Lys454) and three in *Pgi2* (Asn131, Asn324 and Gly425). All these codons involve predicted amino acid substitutions that vary in residue polarity and hydrophobicity, apart from Asn63 in *Gapdh*, where only hydrophobicity is altered (Figure 2). The two *Pgi* paralogues share a similar pattern of BEB posterior probabilities (Figure 3), with regions of high posterior probability (P > 0.75) being predominately located in disordered regions of the protein secondary structure (*Pgi1* = 3 out of 4; *Pgi2* = 7 out of 11; Figure 3) as opposed to well-structured α-helices (*Pgi1* = 1 out of 4; *Pgi2* = 4 out of 11) or β-strands (*Pgi1* = 0 out of 4; *Pgi2* = 0 out of 11). Disordered regions are defined as areas that in isolation do not fold into well-defined 3D structures and conversely sample a large portion of the available conformational space, including features such as loops and extended domains [37]. In other arthropod species, sites identified as either under positive selection, balancing selection, associated with fitness differences or segregating between species in *Pgi* also predominately occur within these disordered regions (*Colias* sp.: 18 out of 27 [17]; *Melitaea cinixia*: 4 out of 5 [18]; *Tigriopus californicus*: 4 out of 6 [38]. Similar patterns of high posterior probabilities of positive selection are seen among paralogues and orthologues (Figure 3). There is direct overlap between Val414 in *Pgi1*, which is under positive selection (P = 0.999), and the homologous site in *Pgi2* (P = 0.836). Additionally, Asn131, which is under positive selection in *Pgi2* (P = 0.98) is homologous to a segregating site between *Colias* sp. (Gly128) [17].

**Table 2:** Amino acid sites identified as being under positive selection

**Gene**	Site*	**BEB**	**ω M8 (**^**+**^**/**_**− **_**SE)**	**Amino acids**
*Gapdh*	63	0.993	1.794 (0.821)	C,N,S
*Pgi1*	414	0.999	2.532 (0.809)	V,I,K,T
	454	0.999	2.533 (0.807)	K,R,S,N,A,T,E
*Pgi2*	131	0.981	1.498 (0.148)	A,K,T,V
	324	0.975	1.493 (0.162)	V,A,T,M
	425	0.955	1.474 (0.208)	G,R

Inferred using the M8 model in *Gapdh*, *Pgi1* and *Pgi2* from Lanceocercata species.

* = Amino acid site in the full length *Micrarchus nov. sp. 2* sequences. SE = standard error. BEB = Bayes Empirical Bayes posterior probability.

**Figure 4:**
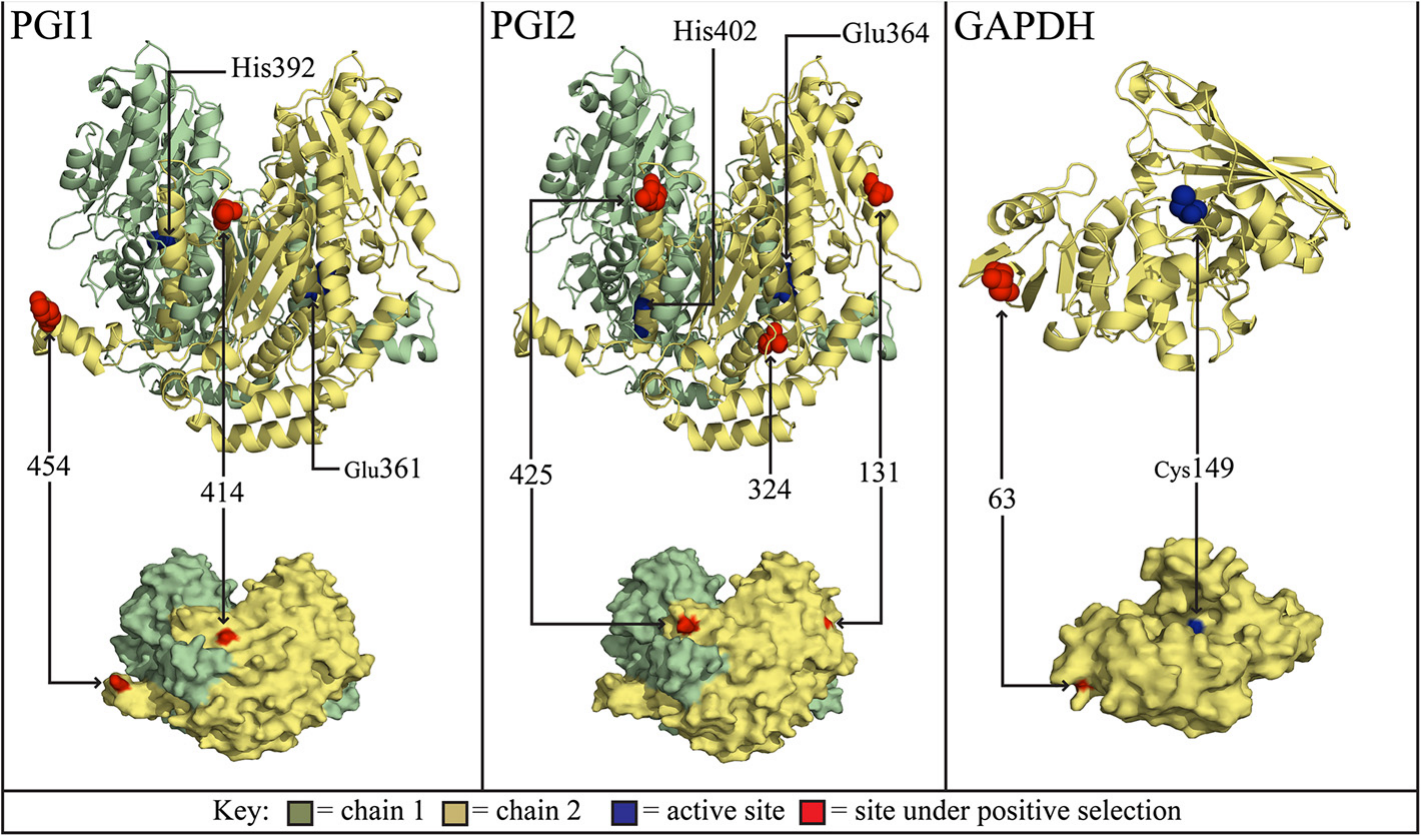
**PGI and GAPDH structural models.** Structural model highlighting the location of amino acid sites under selection and active sites of Lanceocercata stick insect PGI1, PGI2 and GAPDH enzymes.

High resolution crystal structures of the glycolytic enzymes are available, allowing the location of codons identified as under positive selection by the BEB analysis to be visualised in a structural context. Predicted structural models of full length *Micrarchus* nov. sp. 2 protein sequences were generated using SWISS-MODEL [39]. PGI1 and PGI2 were modelled against the pig PGI structure [40] (1GZD; *E* = 0; sequence identity = 70 & 66% respectively), whereas *Gapdh* was modelled against the spiny blue lobster GAPDH structure [41] (1DSS; *E* = 1.71e^-146^; sequence identity = 81%). Five of six residues under positive selection in these enzymes are located on the surface of the protein (Figure 4). None of the sites under positive selection are closely associated with the conserved active site residues (Figure 4).

## Discussion

Here we infer positive selection within the glycolytic pathway of Australasian Lanceocerata stick insects encompassing a wide diversity of lifestyles and habitats. The genes under positive selection are not key metabolic control steps regulating flux through the glycolytic pathway. Across the pathway, *Pgi* and *Gapdh* exhibit the strongest signal for positive selection using the conservative codon-based likelihood methods. This may be attributed to their positions in the glycolysis pathway and association with branch points diverting flux through the pentose phosphate pathway (PPP) (Figure 1).

The PPP synthesises NADPH for lipid biosynthesis and to neutralise reactive oxygen species accumulated during periods of oxidative stress [1]. *Pgi* allelic variants and GAPDH inactivation are respectively known to differentially regulate flux through the PPP in the sea anemone *Metridium senile*[2] and *C. elegans*[1]. Excess NADPH synthesised by the PPP may play an important role in adaptation to novel stressful life histories by allowing insects either to exploit different energy storage pools, or to compensate for increased oxidative stress, or both. Increased lipid biosynthesis is documented in overwintering insects [26] and winged morphs of *Gryllus firmus*[25]. Insects are also particularly susceptible to oxidative stress due to: (i) their tracheal respiratory system; (ii) herbivorous diets being rich in pro-oxidants [42]; (iii) extreme demand for oxygen during flight [22]; and (iv) exposure to low-temperature [23].

The amino acid substitutions at the sites inferred to be under positive selection do not discretely partition among different life history strategies. The lack of a clear pattern may in part be due to the complexity of their life-history traits. For example, alpine species may experience selection for vastly different metabolic regimes depending on whether they are freeze tolerant or freeze avoidant [43]. Understanding how adaptation to new stressful lifestyles alters flux through both the glycolytic and PPP will require integrated biochemical enzyme assays in relation to sites under positive selection [44,45], and will ultimately indicate the relative importance of maintaining energy production against exploiting the different energy storage pools and reducing the cost of stress induced oxidative damage in adaptation.

The continued association of *Pgi* with an array of fitness measures has led to the suggestion that this locus could be used as an adaptive marker in arthropods [13]. However, the occurrence of paralogues affects its practical implementation using traditional PCR-based methods, at least in the Euphasmatodea. Gene duplications may become fixed if there is existing balancing selection, as the fixation of both original alleles removes the possibility of the deleterious occurrence of homozygotes at the original locus [46]. The *Pgi* duplications in Lanceocercata are possibly a consequence of existing balancing selection preceding subsequent gene duplication. Gene duplications may eventually lead to subfunctionalisation of tissue-specific forms of the protein, as seen in teleost fish *Pgi*[47] and *Tpi*[48].

The positive selection acting on *Pgi1* and *Pgi2* results in changes to the polarity and hydrophobicity of the translated amino acids. The charge-changing amino acid substitutions in the stick insect PGI proteins may cause biochemical and fitness differences similar to those observed in *Colias* PGI [17,18]. In both stick insects and *Colias*, a majority of these sites occur in disordered regions of the PGI secondary structure, in regions that are more conformationally-flexible than the α-helices and β-strands. These intrinsically-disordered protein regions are thought to tolerate increased genetic variation and are important for maintaining adaptive potential [37,49]. Furthermore, protein function is maintained by purifying selection against internal changes that may disrupt enzyme function, restricting positive selection to amino acids exposed to the surface of the protein’s three dimensional structure [50]. Relatively few modifications are required for environment-specific adaptations to develop, as seen in vertebrate rhodopsin where 94% of amino acid sites are evolving neutrally [36], and in *Mytilus* mussels where temperature adaptation in isocitrate dehydrogenase is driven by two amino acid substitutions [51]. While surface modifications can alter catalytic performance of enzymes, as seen in lactate dehydrogenase [52], they are more likely to affect a protein’s thermal stability [53]. A similar pattern of positive selection across the overlapping protein coding sequence is observed in the *Pgi* paralogues investigated here. There is also correspondence in the position of sites under positive directional selection in the stick insects and those identified as either under positive selection, associated with fitness differences or segregating between other arthropod species ([18,38,54]; Figure 3). A majority of the *Pgi* coding sequence is conserved by strong purifying selection maintaining protein function. However, positive selection appears to be able to act on specific portions of the *Pgi* gene that are: (i) intrinsically disordered regions of the protein structure where genetic variation is maintained; and (ii) surface residues that can influence enzyme conformation and efficiency. Modifications within these convergent windows of positive selection are presumably responsible for driving enzyme properties towards local optima in response to a host of species-specific adaptations.

## Conclusion

These results from Lanceocercata stick insects illustrate that adaptations within metabolic pathways may be a delicate trade-off between maintaining energy production, optimally exploiting different energy pools, and reducing the effects of increased oxidative stress in the system. Maintaining this balance affects organisms regardless of the adaptation, and selection likely targets enzymes at branch points due to their pleiotropic effects on flux through metabolic pathways. The complexity of enzyme and pathway interactions requires further integrative studies to understand how the metabolic network evolves as a whole.

## Methods

Lanceocercata specimens were collected from Australia, New Caledonia and New Zealand (Additional file 1: Table S3). Samples were preserved in ethanol and stored at –20°C or directly snap frozen and stored at –80°C before RNA extraction. Total RNA was extracted from leg muscle using the RNeasy Mini kit (Qiagen) from 55 individuals representing 29 species. First strand cDNA synthesis was random hexamer primed using the SuperScript III First Strand Kit (Invitrogen) following the manufacturer’s protocol. cDNA libraries were subsequently amplified used 5 μL first strand cDNA, 5 μL of 2 mM dNTP (Roche), 10 μL of 10X FastStart Taq DNA Polymerase PCR Buffer with MgCl2 (Roche),1 μL of 15 mM MgCl2 (Roche), 2 U FastStart Taq DNA Polymerase (Roche) and 5 μL of 25 μM random hexamer in a total volume of 53 μL. Amplifications were performed on a GeneAmp PCR system 9700 thermal cycler (Applied Biosystems) using the following parameters: 5 min at 95°C, 3 min at 50°C , 40 sec 72°C; 40 cycles of 40 sec at 94°C,40 sec at 65°C and 40 sec at 72°C; and 10 min at 72°C.

### Sequencing of glycolysis genes

Primers to amplify partial gene sequences for 19 genes were designed from three 454 *de novo* transcriptomes of three New Zealand species (*Micrarchus* nov. sp. 2 [31], *Niveaphamsa annulata* and *Clitarchus hookeri*) using Primer3 [55] in Geneious v.5.6 [56]. The *N. annulata* and *C. hookeri* transcriptomes were constructed from previously published methods [31] and consisted of 276 K reads from eight individuals and 235 K reads from six individuals, respectively (NCBI SRA submission SRA061479 & SRA061480). Primers were designed to amplify parts of ten gene transcripts (*Eno*, *Galm*, *Gapdh*, *Pdh*, *Pgi1*, *Pgi2*, *Pgk*, *Pglym*, *Pyk*, and *Tpi*) distributed across the glycolytic pathway. In addition, six nuclear protein coding genes with a variety of cellular functions (*Calr*, *Ef1a*, *Flnc*, *Gsk3b*, *Mer* and *Sord*), two mitochondrial genes (*COI*, *COII*), and 28S rDNA were sequenced to assist with phylogeny reconstruction. Target genes were PCR amplified with reactions consisting of approximately 5 ng cDNA, 2.5 μL of 2 mM dNTP (Roche), 1 μL of 10 μM BSA (Sigma-Aldrich), 2.5 μL of 10X FastStart Taq DNA Polymerase PCR Buffer with MgCl2 (Roche), 1.5 U FastStart Taq DNA Polymerase (Roche) and 7.25 pmol of the forward and reverse primers (Sigma-Aldrich) in a total volume of 25 μL. Amplifications were performed on a GeneAmp PCR system 9700 thermal cycler (Applied Biosystems) using the following parameters: 5 min at 95°C; 35 cycles of 1 min at 94°C,1 min at primer specific annealing temperature and 1 min at 72°C; and 10 min at 72°C (Primer sequences and annealing temperature in Additional file 1: Table S2). Successful amplification was assessed by gel electrophoresis on 1% agarose gels containing 0.5 mg mL^–1^ ethidium bromide.

PCR products were sequenced using BigDye Terminator Cycle Sequencing Ready Reaction Mix v3.1 (Applied Biosystems). Cycle sequencing products were cleaned using the BigDye Xterminator Purification Kit (Applied Biosystems) and analysed in both directions on the ABI Prism 3100 Genetic Analyzer (Applied Biosystems). Sequences were subsequently cleaned, trimmed and aligned using Geneious v.5.6. To verify nucleotide substitutions were not a result of polymerase errors, the generated sequences were compared with the High-Throughput Sequencing generated data; and intraspecifically if multiple samples were available for a species. Three core glycolytic enzymes were not characterised, due to multiple expressed copies (hexokinase) or insufficient sequence information in the original transcriptomes (phosphofructokinase and aldose). Sequences were submitted to the NCBI Genbank repository (accession KF383428 to KF383954).

Subsequent sequence analyses only used one sequence per species selected on the tiered criteria of: sequence length; fewest variable nonsynonymous sites; fewest synonymous sites; greatest agreement to consensus; and finally, using the individual selected for a majority of other genes. If necessary, haplotype reconstruction of polymorphic sequences was performed using SeqPHASE [57] and PHASE v2.1 [58,59] (probability of > 90%) prior to calculating descriptive statistic in DnaSP v.5 [60]. Substitution models for nucleotide and amino acid alignments were selected using the corrected Akaike information criterion [61] from jModelTest v.0.1.1 [62,63] and ProtTest3 v.3.2 [63,64] respectively. A Bayesian phylogeny for the 15,034 bp partitioned alignment using MrBayes v.3.2 [65,66] run for 10,000,000 generations. Additional phylogeny nodal support values were obtained by maximum likelihood (100 bootstraps) in Garli v.2.0 [67].

### 4.5.2 Inferring selection

To screen for positive selection in the ten glycolysis protein coding genes, ω was estimated by maximum likelihood using site models implemented in the CODEML program of PAML v.4.5 [33]. The models implemented (M0, M1a, M2a, M3, M7, M8 and M8a) are extensively described elsewhere [33]. Complex models (M1a, M2a, M3, M7, M8, M8a) permit more than one category of ω, allowing individual codons to be identified as under positive selection (>1) when the average ω across the whole gene indicates purifying selection (<1). Likelihood ratio tests (LRTs) between models where ω is allowed to vary above one, and the associated null models where ω is fixed at one, allow inference on the selective pressure acting on the sequence [33]. Codons under positive selection were identified using BEB method under the M8 model. Secondary protein structures were predicted using PSIPRED V.3.3 [68]. Three-dimensional structural modelling of genes identified as under positive selection was performed using SWISS-MODEL [39] and visualised in PyMOL v.0.99 [69].

## Availability of supporting data

The data sets supporting the results of this article are available in the National Center for Biotechnology Information GenBank (KF383428 to KF383954) and Sequence Read Archive (SRA061479 and SRA061480) repositories.

## Competing interests

The authors declare that they have no competing interests.

## Authors’ contribution

LTD and GT sequenced target genes; AD provided additional transcriptome data; LTD Preformed Selection analyses; LTD, AD, BJS, RDN and TRB conceived of the study, and participated in its design and coordination and helped to draft the manuscript. All authors read and approved the final manuscript.

## Supplementary Material

Additional file 1: Table S1 Likelihood parameter values for site models. **Table S2.** Primer sequences and annealing temperatures. **Table S3.** Species samples collection details. **Figure S1.** Bayesian arthropod PGI phylogeny. **Figure S2.** Amino acid alignment of all three PGI paralogues. Click here for file
