# Supplementary material for: Positive selection in glycolysis among Australasian stick insects
Source: BMC Evol Biol. 2013 Sep 30;13:215. doi: 10.1186/1471-2148-13-215 (PMC3850572; doi:10.1186/1471-2148-13-215)
Supplement: Additional file 1: Table S1 — Likelihood parameter values for site models. Table S2. Primer sequences and annealing temperatures. Table S3. Species samples collection details. Figure S1. Bayesian arthropod PGI phylogeny. Figure S2. Amino acid alignment of all three PGI paralogues. [file 1471-2148-13-215-S1.docx]

**Supplementary Material**

**Supplementary Table 1a-j:** Likelihood parameter values for site and branch specific models for ten enzymes associated with glycolysis in Lanceocercata.

**Supplementary Table 1a:** *Eno* (Enolase)

| **Model** | ***l*** | **dN/dS** | **Estimates of parameters** |
| --- | --- | --- | --- |
| **M0** | -3796.50 | 0.110 | *ω* = 0.10964 |
| **M1a** | -3734.32 | 0.143 | *p_0_* = 0.88930, (*p_1_* = 0.11070) |
| **M2a** | -3734.32 | 0.143 | *p_0_* = 0.88930, *p_1_* = 0.11070, (*p_2_* = 0.00000), *ω_2_* = 30.50253 |
| **M3** | -3730.75 | 0.121 | *p_0_* = 0.82411, *p_1_* = 0.14927, (*p_2_* = 0.02632) *ω_0_* = 0.01994, *ω_1_* = 0.47404, *ω_2_* = 1.28698 |
| **M7** | -3731.79 | 0.120 | *p* = 0.11579, *q* = 0.82851 |
| **M8** | -3730.84 | 0.121 | *p_0_* = 0.94785, (*p_1_* = 0.05215) *p* = 0.17392, *q* = 2.14520, *ω* = 1.05350 |
| **M8a** | -3730.85 | 0.121 | *p_0_* = 0.94016, (*p_1_* = 0.05984) *p* = 0.18256, *q* = 2.42855, *ω* = 1.00000 |

**Supplementary Table 1b:** *Galm* (Aldose 1-epimerase)

| **Model** | ***l*** | **dN/dS** | **Estimates of parameters** |
| --- | --- | --- | --- |
| **M0** | -3555.43 | 0.155 | *ω* = 0.1552 |
| **M1a** | -3454.87 | 0.188 | *p_0_* = 0.84695, (*p_1_* = 0.15305) |
| **M2a** | -3454.87 | 0.188 | *p_0_* = 0.84695, *p_1_* = 0.04145, (*p_2_* = 0.09486), *ω_2_* = 0.05819 |
| **M3** | -3449.96 | 0.172 | *p_0_* = 0.71804, *p_1_* = 0.19258, (*p_2_* = 0.08938) *ω_0_* = 0.01639, *ω_1_* = 0.28882, *ω_2_* = 1.17527 |
| **M7** | -3453.18 | 0.162 | *p* = 0.12494, *q* = 0.64155 |
| **M8** | -3450.14 | 0.172 | *p_0_* = 0.91168, (*p_1_* = 0.08832) *p* = 0.24183, *q* = 2.79451, *ω* = 1.17181 |
| **M8a** | -3450.48 | 0.164 | *p_0_* = 0.89364, (*p_1_* = 0.10636) *p* = 0.26738, *q* = 3.64572, *ω* = 1.00000 |

**Supplementary Table 1c:** *Gapdh* (Glyceraldehyde 3-phosphate dehydrogenase)

| **Model** | ***l*** | **dN/dS** | **Estimates of parameters** |
| --- | --- | --- | --- |
| **M0** | -3779.93 | 0.043 | *ω* = 0.04280 |
| **M1a** | -3719.72 | 0.055 | *p_0_* = 0.95800, (*p_1_* = 0.04200) |
| **M2a** | -3719.05 | 0.061 | *p_0_* = 0.95855, *p_1_* = 0.03851, (*p_2_* = 0.00294), *ω_2_* = 3.23704 |
| **M3** | -3716.50 | 0.052 | *p_0_* = 0.94120, *p_1_* = 0.05501, (*p_2_* = 0.00379) *ω_0_* = 0.00967, *ω_1_* = 0.56760, *ω_2_* = 3.08936 |
| **M7** | -3722.68 | 0.065 | *p* = 0.04709, *q* = 0.63918 |
| **M8** | -3717.29 | 0.049 | *p_0_* = 0.97641, (*p_1_* = 0.02359) *p* = 0.07744, *q* = 2.70902, *ω* = 1.16462 |
| **M8a** | -3717.41 | 0.048 | *p_0_* = 0.97151, (*p_1_* = 0.02849) *p* = 0.08807, *q* = 3.51764, *ω* = 1.00000 |

**Supplementary Table 1d:** *Pdh* (Pyruvate dehydrogenase)

| **Model** | ***l*** | **dN/dS** | **Estimates of parameters** |
| --- | --- | --- | --- |
| **M0** | -3128.40 | 0.052 | *ω* = 0.05235 |
| **M1a** | -3078.51 | 0.072 | *p_0_* = 0.94248, (*p_1_* = 0.05752) |
| **M2a** | -3078.51 | 0.072 | *p_0_* = 0.94248, *p_1_* = 0.05752, (*p_2_* = 0.00000), *ω_2_* = 25.93450 |
| **M3** | -3076.76 | 0.060 | *p_0_* = 0.65061, *p_1_* = 0.28323, (*p_2_* = 0.06616) *ω_0_* = 0.00000, *ω_1_* = 0.04530, *ω_2_* = 0.71440 |
| **M7** | -3077.75 | 0.066 | *p* = 0.05109, *q* = 0.67726 |
| **M8** | -3077.52 | 0.067 | *p_0_* = 0.95301, (*p_1_* = 0.04699) *p* = 0.11580, *q* = 4.57465, *ω* = 1.00000 |
| **M8a** | -3077.52 | 0.067 | *p_0_* = 0.95301, (*p_1_* = 0.04699) *p* = 0.11580, *q* = 4.57467, *ω* = 1.00000 |

**Supplementary Table 1e:** *Pgi1* (Phosphoglucose isomerase 1)

| **Model** | ***l*** | **dN/dS** | **Estimates of parameters** |
| --- | --- | --- | --- |
| **M0** | -2786.10 | 0.104 | *ω* = 0.10370 |
| **M1a** | -2675.58 | 0.141 | *p_0_* = 0.87532, (*p_1_* = 0.12468) |
| **M2a** | -2664.42 | 0.197 | *p_0_* = 0.87135, *p_1_* = 0.11971, (*p_2_* = 0.00895), *ω_2_* = 6.84299 |
| **M3** | -2657.82 | 0.151 | *p_0_* = 0.83012, *p_1_* = 0.16085, (*p_2_* = 0.00903) *ω_0_* = 0.00744, *ω_1_* = 0.56138, *ω_2_* = 6.04082 |
| **M7** | -2669.64 | 0.126 | *p* = 0.04969, *q* = 0.34623 |
| **M8** | -2656.99 | 0.153 | *p_0_* = 0.99100, (*p_1_* = 0.00900) *p* = 0.07135, *q* = 0.63715, *ω* = 6.08312 |
| **M8a** | -2669.64 | 0.126 | *p_0_* = 0.99999, (*p_1_* = 0.00001) *p* = 0.04969, *q* = 0.34624, *ω* = 1.00000 |

**Supplementary Table 1f:** *Pgi2* (Phosphoglucose isomerase 2)

| **Model** | ***l*** | **dN/dS** | **Estimates of parameters** |
| --- | --- | --- | --- |
| **M0** | -4908.76 | 0.098 | *ω* = 0.09770 |
| **M1a** | -4816.51 | 0.118 | *p_0_* = 0.92193, (*p_1_* = 0.07807) |
| **M2a** | -4816.11 | 0.125 | *p_0_* = 0.92392, *p_1_* = 0.07023, (*p_2_* = 0.00586), *ω_2_* = 2.40926 |
| **M3** | -4800.27 | 0.114 | *p_0_* = 0.66727, *p_1_* = 0.29625, (*p_2_* = 0.03648) *ω_0_* = 0.00000, *ω_1_* = 0.19952, *ω_2_* = 1.50572 |
| **M7** | -4810.17 | 0.110 | *p* = 0.11326, *q* = 0.88951 |
| **M8** | -4801.90 | 0.114 | *p_0_* = 0.96764, (*p_1_* = 0.03236) *p* = 0.20741, *q* = 2.77776, *ω* = 1.57978 |
| **M8a** | -4804.19 | 0.101 | *p_0_* = 0.95066, (*p_1_* = 0.04934) *p* = 0.22868, *q* = 3.67539, *ω* = 1.00000 |

**Supplementary Table 1g:** *Pgk* (Phosphoglycerate kinase)

| **Model** | ***l*** | **dN/dS** | **Estimates of parameters** |
| --- | --- | --- | --- |
| **M0** | -5061.85 | 0.194 | *ω* = 0.19374 |
| **M1a** | -4929.70 | 0.229 | *p_0_* = 0.81155, (*p_1_* = 0.18845) |
| **M2a** | -4929.70 | 0.229 | *p_0_* = 0.81155, *p_1_* = 0.11620, (*p_2_* = 0.07225), *ω_2_* = 1.00000 |
| **M3** | -4920.31 | 0.206 | *p_0_* = 0.56750, *p_1_* = 0.30438, (*p_2_* = 0.12812) *ω_0_* = 0.00000, *ω_1_* = 0.22905, *ω_2_* = 1.06606 |
| **M7** | -4922.92 | 0.196 | *p* = 0.13541, *q* = 0.55380 |
| **M8** | -4921.25 | 0.207 | *p_0_* = 0.91323, (*p_1_* = 0.08677) *p* = 0.19958, *q* = 1.49523, *ω* = 1.18305 |
| **M8a** | -4921.54 | 0.201 | *p_0_* = 0.87279, (*p_1_* = 0.12721) *p* = 0.23192, *q* = 2.38946, *ω* = 1.00000 |

**Supplementary Table 1h:** *Pglym* (Phosphoglycerate mutase)

| **Model** | ***l*** | **dN/dS** | **Estimates of parameters** |
| --- | --- | --- | --- |
| **M0** | -2228.23 | 0.078 | *ω* = 0.07787 |
| **M1a** | -2158.87 | 0.102 | *p_0_* = 0.91321, (*p_1_* = 0.08679) |
| **M2a** | -2158.87 | 0.102 | *p_0_* = 0.91321, *p_1_* = 0.06913, (*p_2_* = 0.01767), *ω_2_* = 1.00000 |
| **M3** | -2151.7 | 0.094 | *p_0_* = 0.81373, *p_1_* = 0.14290, (*p_2_* = 0.04337) *ω_0_* = 0.00000, *ω_1_* = 0.26670, *ω_2_* = 1.27599 |
| **M7** | -2153.78 | 0.119 | *p* = 0.03471, *q* = 0.25567 |
| **M8** | -2152.31 | 0.090 | *p_0_* = 0.95528 (*p_1_* = 0.04472) *p* = 0.05604, *q* = 1.22788, *ω* = 1.24774 |
| **M8a** | -2152.65 | 0.084 | *p_0_* = 0.94481, (*p_1_* = 0.05519) *p* = 0.05049, *q* = 1.24849, *ω* = 1.00000 |

**Supplementary Table 1i:** *Pyk* (Pyruvate kinase)

| **Model** | ***l*** | **dN/dS** | **Estimates of parameters** |
| --- | --- | --- | --- |
| **M0** | -3834.24 | 0.036 | *ω* = 0.03626 |
| **M1a** | -3777.76 | 0.071 | *p_0_* = 0.93970, (*p_1_* = 0.06030) |
| **M2a** | -3777.76 | 0.071 | *p_0_* = 0.93970, *p_1_* = 0.06030, (*p_2_* = 0.00000), *ω_2_* = 13.80843 |
| **M3** | -3761.24 | 0.040 | *p_0_* = 0.61439, *p_1_* = 0.30532, (*p_2_* = 0.08030) *ω_0_* = 0.00006, *ω_1_* = 0.2126, *ω_2_* = 0.41875 |
| **M7** | -3762.33 | 0.045 | *p* = 0.05735, *q* = 1.04612 |
| **M8** | -3762.33 | 0.045 | *p_0_* = 0.99999, (*p_1_* = 0.00001) *p* = 0.05736, *q* = 1.04640, *ω* = 1.40148 |
| **M8a** | -3762.33 | 0.045 | *p_0_* = 0.99999, (*p_1_* = 0.00001) *p* = 0.05736, *q* = 1.04634, *ω* = 1.00000 |

**Supplementary Table 1j:** *Tpi* (Triosephosphate isomerase) CT

| **Model** | ***l*** | **dN/dS** | **Estimates of parameters** |
| --- | --- | --- | --- |
| **M0** | -2716.07 | 0.139 | *ω* = 0.13928 |
| **M1a** | -2693.6 | 0.180 | *p_0_* = 0.87314, (*p_1_* = 0.12686) |
| **M2a** | -2693.6 | 0.180 | *p_0_* = 0.87314, *p_1_* = 0.10137, (*p_2_* = 0.02549), *ω_2_* = 1.00000 |
| **M3** | -2689.54 | 0.154 | *p_0_* = 0.67892, *p_1_* = 0.31455, (*p_2_* = 0.00654) *ω_0_* = 0.01154, *ω_1_* = 0.41197, *ω_2_* = 2.57660 |
| **M7** | -2690.41 | 0.153 | *p* = 0.19881, *q* = 1.08056 |
| **M8** | -2689.77 | 0.156 | *p_0_* = 0.99464, (*p_1_* = 0.00536) *p* = 0.22782, *q* = 1.33953, *ω* = 2.63921 |
| **M8a** | -2690.25 | 0.152 | *p_0_* = 0.96492, (*p_1_* = 0.03508) *p* = 0.24364, *q* = 1.70338, *ω* = 1.00000 |

**Supplementary Table 2:** Primer sequences and annealing temperatures. Genes were either amplified in one complete fragment or with the use of internal primers.

| Gene | Primer and Direction | 5'-3' | Annealing |
| --- | --- | --- | --- |
| Aldose 1-epimerase | Galm_313F | ACAATCCGTATTTCGGAGCAACCA | 60 |
|  | Galm_663F | TTCAACTGGGTCCGAGAGCATC | 60 |
|  | Galm_789R | TCAGCTGCGGGAATGCCAGTT | 60 |
|  | Galm_998R | TGCAGCATGCTTCCTGTACGTGT | 60 |
| Calreticulin | Calr_283F | ACCTGGTGGTGCAGTTCACGG | 58 |
|  | Calr_790F | AGAGTGGGAGCCGCCCATGAT | 58 |
|  | Calr_811R | TGCTTGGGCTTCCACTCGCC | 58 |
|  | Calr_1167R | GGCGTGTCTCCTGCTTCTGCC | 58 |
| Cytochrome oxidase I & II | CI-J-2195 | (Simon *et al.,* 1994) | 54 |
|  | TL2-N-3014 | (Simon *et al.,* 1994) | 54 |
|  | TL2-J-3034 | (Simon *et al.,* 1994) | 54 |
|  | TK-N-3785 | (Simon *et al.,* 1994) | 54 |
| Elongation factor 1 alpha | Ef1a-795-F | CCACCTAGCCGTCCCACGGA | 62 |
|  | Ef1a-1496-R | GTCGGCAGGGCAGAGAGCTG | 62 |
| Enolase | Eno_185F | AGCACTACCACGGAAAGGGGGT | 56 |
|  | Eno_10901 | ACCATGGTGCCCCAGCCATT | 56 |
| Filamin-C | Flnc_360F | TCGTACCTTCCCACTGCCCCT | 60 |
|  | Flnc_1301R | TGGCCCACCTGCATGCACTC | 60 |
| Glyceraldehyde 3-phosphate dehydrogenase | Gapdh_446R | TGCCAGGCAGTTGGTGGTGC | 60 |
|  | Gapdh_43F | ATTCGGCCGCATCGGTCGCC | 60 |
|  | Gapdh_338F | TGCTCACTTGGAAGGTGGGGC | 60 |
|  | Gapdh_885R | TGCTGAGGGGAATGCCAGCC | 60 |
| Glycogen synthase kinase 3 beta | Gsk3b_310F | GCGAAGCTATGCGACACTGGGG | 64 |
|  | Gsk3b_1267R | CCGGGGTTGTTGCTCGCAGT | 64 |
|  | Gsk3b_815F | AGCGCTGGGTGTGTTCTGGC | 64 |
|  | Gsk3b_818R | GGGTTGCCCCAGCAGCAGTT | 64 |
| Moesin ezrin radixin homolog | Mer_863F | GCGCATCCTGGCCTTGTGCAT | 60 |
|  | Mer_1676R | GCGCTTCGTGTTGCCCTTGC | 60 |
|  | Mer_1168F | TGAGCTGGAGGAGCGCCAGA | 60 |
|  | Mer_1507R | TCCGCGAGCGTCCTCCTCTC | 60 |
| Phosphoglucose isomerase 1 | Pgi1_924F | GCGCGCACTTCGCTGACAAC | 60 |
|  | Pgi1_1601R | GCTGCTCACCGGCGTCTTGT | 60 |
|  | Pgi1_1143F | GGACTACGCCACAGGCCCCATA | 60 |
|  | Pgi1_1423R | CGACTATGGAGTTGGTGGGGCG | 60 |
| Phosphoglucose isomerase 2 | Pgi2_-6F | GTGAAGACGCGCCCAGCAGT | 70 |
|  | Pgi2_+62R | CCGCCCAATCAGCTCGCAGT | 62 |
|  | Pgi2_702F | CCCAGTCCGCCAAGCAGTGG | 62 |
|  | Pgi2_961R | GCCAGGATCGCCGCCATGTT | 70 |
|  | Pgi2_1149R | CCGGTGTTCCCCACACCACG | 64 |
|  | Pgi2_33F | GTTGTTCAAGCTGCCCAGGCTCACC | 64 |
|  | Pgi2_813F | ATGGACGAGAGGAACATCTTCAGG | 64 |
|  | Pgi2_+104R | CGCACACAATTTTTCTTTGTTACAGCG | 64 |
|  | Pgi2_280F | AGGGACGCCATGTTCGCCG | 64 |
|  | Pgi2_981R | TGTACCACACGCCGAGCAGC | 64 |
|  | Pgi2_833F | TTCTGGGACTGGGTCGGCGG | 64 |
|  | Pgi2_1628R | GGTGGACGGGTCGTGGCTTG | 64 |
| Phosphoglycerate kinase | Pgk_131F | AGCGTATTGTTGCTGCCCTGGA | 62 |
|  | Pgk_1049R | CCACTGCATCCATCAGACCCTTCG | 62 |
|  | Pgk_653F | CGGGGCCAAGGTTGCAGACAA | 62 |
|  | Pgk_673R | TCATCCACGCGGTCGAGCAT | 62 |
| Phosphoglycerate mutase | Pglym_206F | GGCGCAGGACACACTCCAAGC | 60 |
|  | Pglym_695R | CACTGTTTCCTCATCACCCAGGAACT | 60 |
| Pyruvate dehydrogenase | Pdh_236F | AGGCGAATGGAGGCTTCCGC | 60 |
|  | Pdh_969R | GCTCCGCACCGAGGAGCTTG | 60 |
|  | Pdh_458F | CGCGAGGGGTAAAGGTGGCTC | 60 |
|  | Pdh_823R | TGGGTCCCTTGCCACTCGC | 60 |
| Pyruvate kinase | Pyk_149F | ACCATTGGGCCGGCATCACG | 62 |
|  | Pyk_930R | GCTTGCCCGCATGGTTGCAG | 62 |
|  | Pyk_605F | ATGCTGGGCAGCAGGAAGGG | 62 |
|  | Pyk_607R | GGGACTCCGGGCAGGTTCAC | 62 |
| Sorbitol dehydrogenase | Sord_1003R | TTGCCCTTGCCCGTCTTGGC | 66 |
|  | Sord_160F | CCTGGTCAACGGCCGGATCG | 66 |
|  | Sord_208F | CATGGGACACGAGGCGAGCG | 60 |
|  | Sord_955R | AGTTTGAAGTTGTGGGTGAT | 60 |
|  | Sord_581F | GCCAAGGCCATGGGAGCCAG | 60 |
|  | Sord_748R | AGCCTGACGGTGGACTCGGC | 60 |
| Triosephosphate isomerase | Tpi_28F | AGGAAGTTTTGGGTCGGTGGGA | 60 |
|  | Tpi_683R | GCGAAGCTCCACCCACAAGGA | 60 |
|  | Tpi_241F | ACAGGTGAGATCAGCCCAGCAA | 60 |
|  | Tpi_484R | TGGCCCAAACAGGCTCGTATGC | 60 |
| 28s | 28S-356 | (Buckley *et al.* 2008) | 60 |
|  | 28S-1009 | (Buckley *et al.* 2008) | 60 |

**Supplementary Table 3:** Species sample collection details.

| Species | n | Collection Details | ­Coordinates |
| --- | --- | --- | --- |
| *Acanthoxyla* sp. | 1 | NZ, Auckland, Oratia, Waitakere | (-36°55’34”,174°37’02”) |
|  | 1 | NZ , Westland, Okarito, Pack Track to Three Mile Lagoon | (-43°13’17”,170°09’55”) |
| *Anchiale* sp. | 1 | AU, Queensland, Kuranda | (-16°48’55”,145°38’33”) |
| *Argosarchus horridus* | 1 | NZ, Mid Canterbury, Bank’s Peninsula, Okuti Valley | (-43°46’56”,172°49’55”) |
|  | 1 | NZ, Bay of Plenty, Karangahake Gorge | - |
|  | 1 | NZ , Rangitikei, Paengaroa | (-39°38’45”,175°42’37”) |
| *Asprenas impennis* | 1 | NC, Prov. Sud, trail to Plateau de Dogny, | (-21°37', 165°52') |
| *Asprenas sp.* | 1 | NC, Prov. Sud, Col d'Amieu | (-21°33’ S,165°45’) |
| *Asteliaphasma jucundum* | 2 | NZ, Auckland, Kakamatua Park, near Huia, Waitakere | (-37°01’49”,174°31’10”) |
|  | 1 | NZ, Northland, Puketi Forest, Waipapa River Track | (-35°16’41”,173°41’06”) |
| *Canachus sp.* | 2 | NC ,Prov. Sud, Road from Sarraméa to Canala | (-21°35’06”,165°48’56”) |
| *Clitarchus hookeri* | 1 | NZ, Waikato, Lake Karapiro | (-37°56’48”,175°35’35”) |
|  | 1 | NZ, Auckland, Totara Park, Manukau, | (-37°00’04”,174°54’42”) |
| *Clitarchus* nov. sp. 2 | 1 | NZ, Northland, Cape Reinga, Te Paki | (-34°30’03”,172°47’25”) |
| *Clitarchus* nov. sp. 1 | 1 | NZ, Northland, Tawhiti Rahi, Poor Knights Island | (-35°27’19”,174°44’08”) |
| *Cnipsus rachis* | 1 | NC, Prov. Nord, Aoupinié, maintenance hut | (-21°09',165°19’) |
|  | 1 | NC, Prov. Sud, Road from Sarraméa to Canala | (-21°35’06”,165°48’56”) |
| *Ctenomorpha sp.* | 1 | AU, Queensland, Noonbah Station | (-24°05’15”,143°08’41”) |
| Gen. Nov. 1, sp. nov. 1 | 1 | NC, Prov. Sud, Riviére Bleue Pk, | (-22°09’, 166°30’) |
| *Leosthenes sp.* | 1 | NC, Prov. Sud, trail to Plateau de Dogny | (-21°37',165°52') |
| *Micrarchus* nov. sp. 2 (NZAC03009458) | 2 | NZ, Buller, Sewell Peak | (-42°24’19”,171°20’35”) |
|  | 2 | NZ, Nelson, Mt Arthur Trk, ridge above Mt Arthur hut | (-41°11’52”,172°42’45”) |
|  | 1 | NZ, Nelson, Lead Hill eastern slope | (-40°52’25”,172°32’31”) |
|  | 1 | NZ, Nelson, Lake Sylvester Track, Cobb Dam | (-41°06’19”,172°40’57”) |
|  | 1 | NZ, Nelson, Mt Robert | (-41°50’03”,172°48’40”) |
|  | 1 | NZ, Nelson, Denniston Saddle, Whareatea Mine | (-41°46’02”,171°47’15”) |
| *Micrarchus hystriculeus* | 2 | NZ , Rangitikei, Paengaroa | (-39°38’45”,175°42’37”) |
| *Micrarchus* nov. sp. 1 (NZAC03000433) | 2 | NZ, Kaikoura, Puhi Puhi Reserve | (-42°14’22”,173°45’10”) |
| *Micrarchus* nov. sp. 3 (NZAC03000053) | 2 | NZ , Marlborough sounds, Stephens Island, Queens Beach | (-40°40’15”,174°00’15”) |
| *Microcanachus sp.* | 1 | NC, Prov. Sud, Riviére Bleue Park | (-22°09’,166°30) |
| *Niveaphasma annulata* | 1 | NZ , Mackenzie, Lake Ohau Ski Field Road | (-44°14’56”,169°47’48”) |
|  | 1 | NZ, Fjordland, Lk Monowai Road | (-45°48’29”,167°31’32”) |
|  | 1 | NZ, Central otago, The Remarkables, Rastus Burn Road | (-45°01’47”,168°47’09”) |
|  | 1 | NZ , Southland, Old Coach Rd, Papatowai, The Catlins | (-46°33’09”,169°28’29”) |
| Gen. Nov. 2 sp. nov. 1 | 2 | NC, Prov. Nord, Aoupinié, maintenance hut | (-21°09',165°19’) |
| *Pseudoclitarchus sentus* | 1 | NZ, Great Island, Three Kings Islands | (-34°09’34”,172°08’10”) |
| *Spinotectarchus acornutus* | 2 | NZ, Northland, Kohuranaki Reserve | (-34°29’57”,172°52’21”) |
|  | 1 | NZ, Northland, East Herekino, Kaitaia Walkway | (-35°09’47”,173°16’09”) |
| *Tectarchus huttoni* | 1 | NZ, Wellington, Akatarawa Saddle, Tararua Forest | (-40°56’55”,175°06’39”) |
|  | 1 | NZ, Wellington, Kapiti Island, Wilkinson Track | (-40°51’36”,174°55’25”) |
| *Tectarchus ovobessus* | 2 | NZ , Rangitikei, Paengaroa | (-39°38’45”,175°42’37”) |
| *Tectarchus salebrosus* | 1 | NZ, Christchurch, Kennedy’s bush | (-43°37’59”,172°36’25”) |
| *Tepakiphasma ngatikuri* | 1 | NZ , Northland, Kauri Bush | (-34°30’03”,172°47’25”) |
| *Tropidoderus* sp. 1 | 1 | AU, Queensland | - |
| *Tropidoderus* sp. 2 | 1 | AU, Queensland | - |

NZ = New Zealand, Au = Australia, NC = New Caledonia, n = number sequenced.

New Zealand Arthropod Collection (NZAC) code provided for vouchers specimens for undescribed species

for undescribed NC species information refer to Buckley *et. al.* (2010) *Syst Ent* 35:207-225

**Supplementary Figure Legends**

**Supplementary Figure 1:** Bayesian arthropod PGI phylogeny constructed using full length amino acid sequences (alignment = 574 amino acids). Duplications in the Lancerocercata stick insects are highlighted. Phylogeny constructed using MrBayes: 0.5 million generations; average deviation of spilt frequencies < 0.01; WAG amino acid substitution model with Gamma; scale indicates number of substitutions per site. Full length sequences from NCBI GenBank (Accession numbers after species name). *Timema* sequence generated from the published transcriptome: Comeault *et al.* (2012) *Mol Ecol Res* 12: 549-561.

**Supplementary Figure 2:** Amino acid alignment of all three PGI paralogs in *Clitarchus hookeri* (CLH), *Niveaphasma annulata* (NIV) and *Micrarchus* nov. sp. 2 (MI2) transcriptomes. Sites identified as under positive selection across the Lanceocercata (posterior probability > 0.95) using the M8 substitution model and BEB analysis implemented in the CODEML program of the PAML v.4.5 package highlighted in yellow.

**
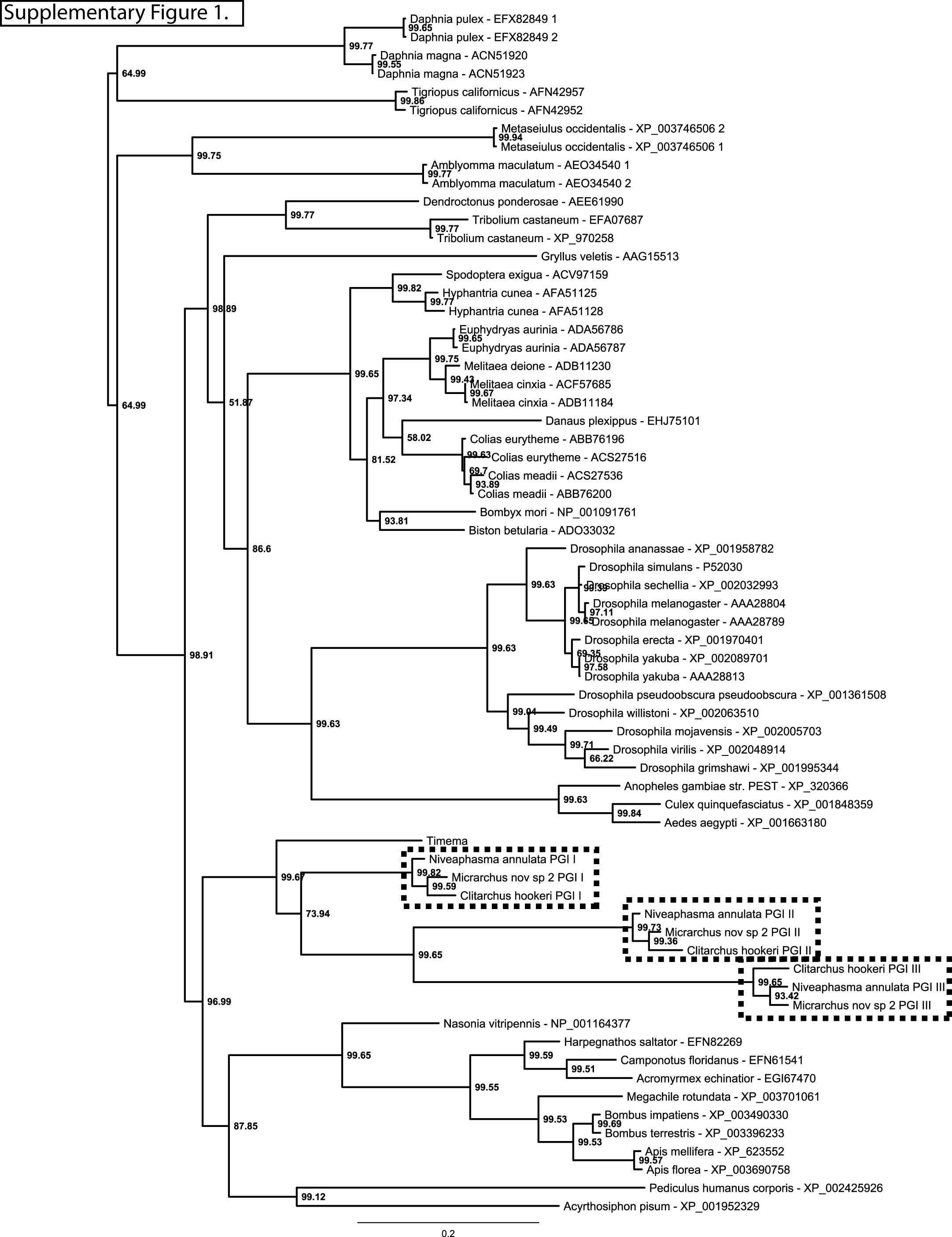
**

**
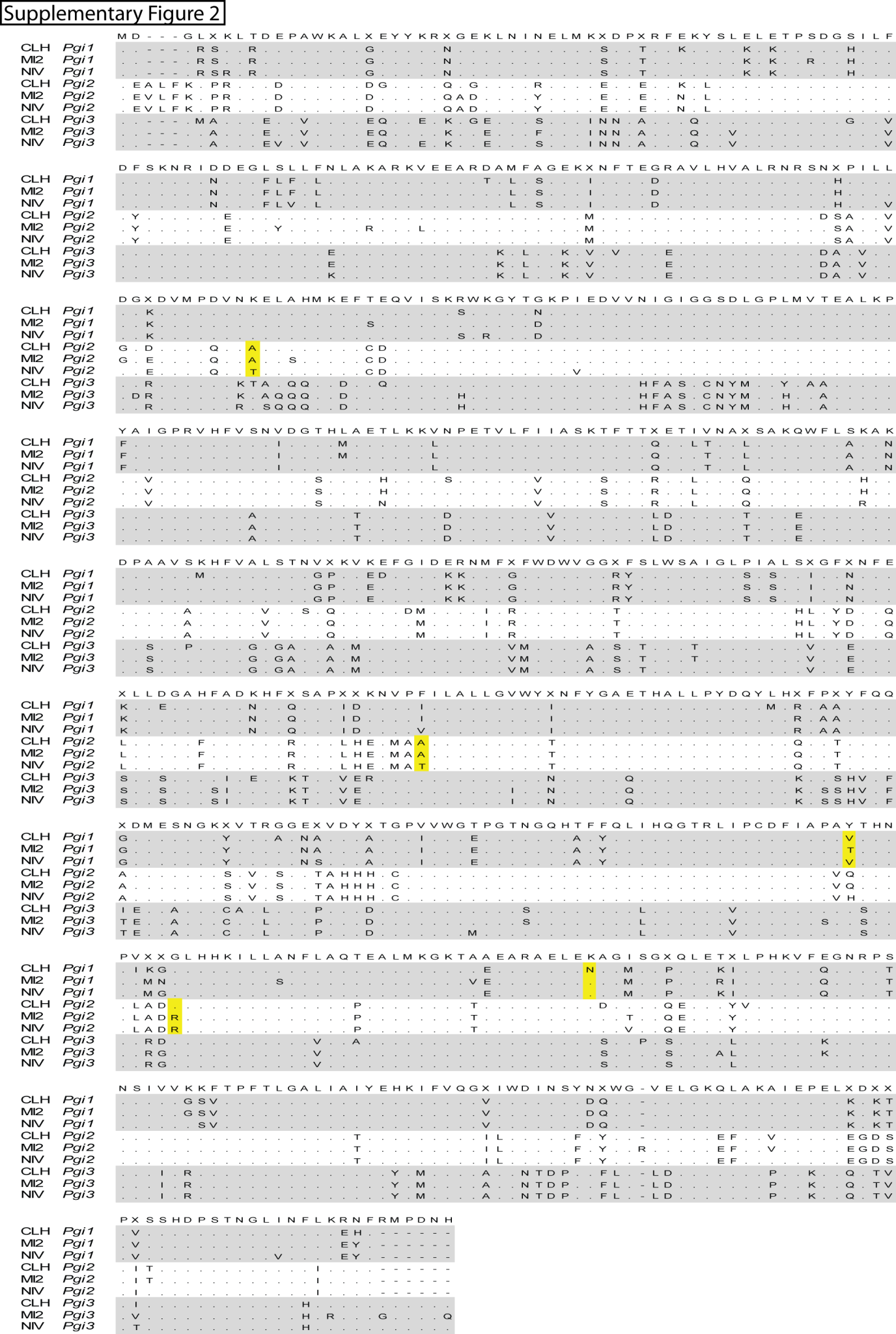
**
